# Enhanced Mobility in Suspended Chemical Vapor-Deposited Graphene Field-Effect Devices in Ambient Conditions

**DOI:** 10.1021/acsami.3c04012

**Published:** 2023-07-25

**Authors:** Kishan Thodkar, Fabian Gramm

**Affiliations:** † Micro- & Nanosystems, Department of Mechanical & Process Engineering, Tannenstrasse 3, ETH Zurich, 8052 Zurich, Switzerland; ‡ ScopeM, Otto-Stern-Weg 3, ETH Zurich, 8093 Zurich, Switzerland

**Keywords:** chemical vapor deposition (CVD), suspended graphene, mobility, scattering, current annealing

## Abstract

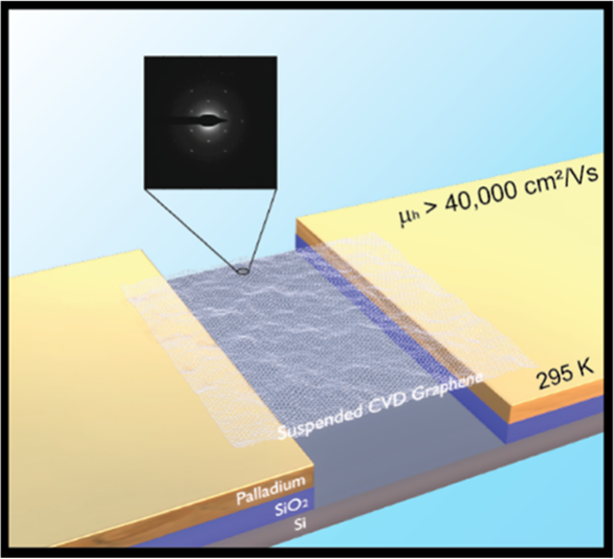

High-field-effect mobility and the two-dimensional nature of graphene films make it an interesting material for developing sensing applications with high sensitivity and low power consumption. The chemical vapor deposition process allows for producing high-quality graphene films in a scalable manner. Considering the significant impact of the underlying substrate on the graphene device performance, methods to enhance the field-effect mobility are highly desired. This work demonstrates a simplified fabrication process to develop suspended, two-terminal chemical vapor deposition (CVD) graphene devices with enhanced field-effect mobility operating at room temperature. Enhanced hole field-effect mobility of up to ∼4.8 × 10^4^ cm^2^/Vs and average hole mobility >1 × 10^4^ cm^2^/Vs across all of the devices is demonstrated. A gradual increase in the width of the graphene device resulted in the increase of the full width at half-maximum (FWHM) of field-effect characteristics and a decrease in the field-effect mobility. Our work presents a simplified fabrication approach to realize high-mobility suspended CVD graphene devices, beneficial for developing CVD graphene-related applications.

## Introduction

Single-layer graphene films offer a unique combination of electronic, mechanical, thermal, and optical properties.^1^ Graphene film consists of sp^2^-hybridized carbon atoms arranged in a hexagonal lattice. It is unique for its linear energy-momentum relation with the conduction and valence band intersecting with zero band gap.^2^ Ballistic charge carrier transport exceeding several micrometers has been demonstrated in graphene devices. Such unique electronic transport properties are beneficial for exploring novel applications such as ballistic electronic devices, rectifiers with high voltage responsivity, graphene-based Josephson junctions in quantum circuits, negative refraction effects, and quantum metrology.^3−9^

Given the unique properties of graphene films, there is an immense interest in large-scale production methods and the commercialization of graphene-based applications. Graphene production techniques have seen significant progress during the last decade, focusing on production methods, the quality of synthetic graphene films, and the integration of graphene films.^10−13^ Chemical vapor deposition (CVD) and epitaxial growth techniques are the two widely utilized techniques to produce high-quality synthetic graphene films on an industrial scale.^10^ Improving the intrinsic quality of synthetic graphene films has been widely studied. “Quality” herein refers to performance metrics such as field-effect mobility, sheet resistance, crystallinity, layer thickness, and the defect-free nature of graphene films. Many studies reported the growth methods of single-crystalline CVD graphene (CVDG) films on large-area copper foils.^11,14−17^ This is promising for realizing wafer-scale production of high-performance CVDG applications. In addition, unique graphene integration methods to “transfer” the graphene films from their growth substrates to target substrates have been widely explored. Some of the well-known transfer methods are polymer-assisted wet etching (WE), electrochemical delamination (ED), lamination-assisted (LA), and dry transfer (DT).^13,18−21^

Such transfer methods play a vital role in determining the performance metrics of the CVDG devices and their applications. Here, we present our study on suspended CVD graphene devices with high-field-effect mobility operating in ambient conditions. Our work utilizes standard cleanroom fabrication processes and the CVD method to fabricate the suspended CVDG devices. We characterize the suspended CVDG devices in ambient conditions and study the influence of increasing CVDG film width on mobility and impurity scattering. We demonstrate high-field-effect mobility of up to ∼4.8 × 10^4^ cm^2^/Vs, and an average hole mobility of >10^4^ cm^2^/Vs across all devices can be achieved in suspended CVDG devices operating at room temperature, realized by standard cleanroom processing methods.

## Results and Discussion

Several graphene device fabrication approaches have been explored over the last few years. The exfoliation method was the first approach to demonstrate the successful isolation of graphene and electrical transport in the material. CVD and epitaxial approaches were later identified as viable methods to produce graphene films on a larger scale beyond the limits of exfoliated graphene flake size. In addition, several device architectures have been explored with a focus on boosting graphene device performance. Three prominent device architectures are (i) on-substrate (type 1), (ii) encapsulated (type 2), and (iii) suspended (type 3). Charge transport in “type 1” graphene devices fabricated on silicon dioxide (SiO_2_) is heavily influenced by the charge traps and corrugations in the oxide layer, thereby limiting their field-effect mobility significantly.^22^ Type 2 and type 3 approaches have explored methods to isolate the graphene film from the oxide. Type 2 approach is focused on encapsulating graphene films within multilayer hexagonal boron nitride (hBN) films.^20,23,24^

Type 3 approaches have explored “suspending” the graphene film, wherein the graphene film is supported at the contact region and freely suspended across the channel region.^25−29^ Prominent performance metrics such as field-effect mobility (μ), charge neutrality point (CNP), full width at half-maximum of the field-effect characteristics (Δ*n*), residual charge carrier density, and minimum conductivity at the CNP provide valuable insights into the graphene device performance. Type 2 and 3 graphene devices significantly improve such performance metrics compared to type 1 devices.^20,23−29^

Charge impurity scattering, defects, grain boundaries, artifacts (folds, wrinkles), and temperature significantly influence the field-effect mobility (μ) of graphene films. Many studies have shown the influence of charge impurity scattering in graphene films. Adam et al. reported a carrier transport theory using self-consistent random phase approximation (RPA)-Boltzmann transport for impurity scattering in graphene.^30^ Chen et al. reported on the influence of depositing potassium atoms on the conductivity of a graphene device, resulting in decreasing field-effect mobility due to charged impurity scattering.^31^ Gosling et al. reported on broadening of the field-effect characteristic due to increasing impurity density on the graphene layer and thereby decreasing mobility.^32^ Exploring the influence of charge impurity scattering in graphene devices helps validate the quality of graphene devices and their fabrication approaches.

Graphene synthesis via chemical vapor deposition is a scalable method to produce graphene films on a large scale. The CVD method allows for synthesizing graphene films on various growth substrates and layer thicknesses. We synthesize graphene on commercially available copper foils (thickness ∼25 μm) using CVD. Polymer-assisted wet etching (WE) process was used to transfer CVDG film onto the target substrate. The WE process consists of three steps.

**Figure 1:**
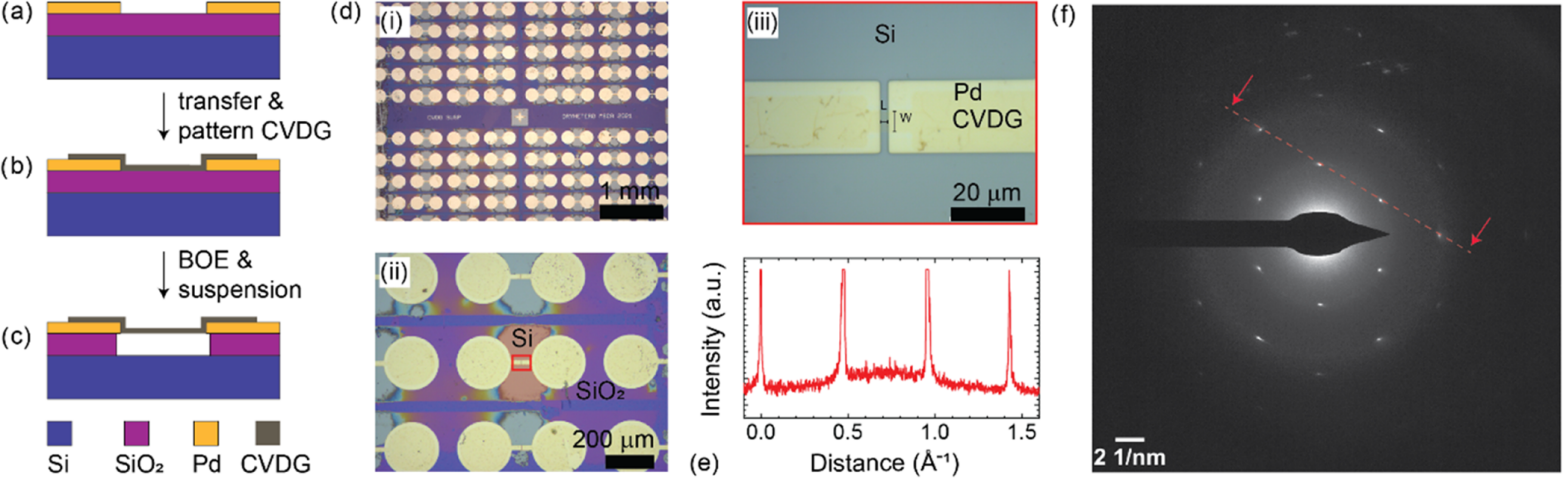
Schematic of the suspended CVDG fabrication process overview consisting of (a) preparation of substrate with contact electrodes (Cr/Pd), (b) transfer and patterning of CVDG, and (c) suspension of CVDG. (d) (i) Optical image of the suspended CVDG device array, (ii) magnified image of the devices with the silicon region highlighted in red, and (iii) optical image of the device area (LxW ∼2 μm x 4 μm) highlighted in the red box in (ii). (e) Intensity line profile of the red dotted line in Figure 1f and (f) selected area electron diffraction image (SAED) of a CVD graphene film on copper foil. Note: The SAED characterization of CVD graphene film was performed on a custom transmission electron microscopy (TEM) grid.

First, a polymer film (poly(methyl methacrylate,) PMMA) was adhered to CVDG on copper (PMMA/CVDG/Cu) using spin coating. Second, the growth substrate wet etching was performed. Third, the PMMA/CVDG layer was transferred to deionized water (DIW) to reduce contamination from etchant exposure. The free-floating PMMA/CVDG layer on the DIW surface is picked up using the preprocessed and structured target substrate (see: Figure 1a) and let to dry. After the drying step, the PMMA layer was dissolved in solvents, and the CVDG layer was transferred onto the target substrate.

Charge traps in the oxide surface and scattering due to the surface phonons at the SiO_2_ interface are attributed to impurity density ∼ >10^11^ cm^–2^, influencing the charge transport in graphene films.^33^ Methods to isolate graphene films is a robust approach to improve device performance. Type 2 approach requires high-quality, exfoliated hBN films for graphene encapsulation. However, the type 3 approach relies on suspending the graphene films such that the field-effect region does not interface with the oxide surface. Considering the feasibility of the CVD process, we present a simplified fabrication approach to realize type 3 graphene devices using standard microfabrication methods.

A schematic description of the suspended CVDG device fabrication is presented in Figure 1a–c. The substrate consists of silicon dioxide (300 nm)/silicon (∼525 μm). First, substrates with the patterned contact electrodes composed of 5 nm chromium/50 nm palladium are prepared using photolithography and thermal evaporation. Second, the CVD graphene/PMMA layer was transferred onto the substrate using the PMMA-assisted wet etching technique. The PMMA layer was dissolved in acetone, and the sample was rinsed in isopropyl alcohol (IPA) and blow-dried in nitrogen airflow. The two-terminal CVDG device structures are patterned using photolithography and oxygen plasma. To suspend the CVDG devices, a buffered oxide etch (BOE) process was used to etch the SiO_2_ layer. Next, a critical point drying (CPD) process was performed to suspend the CVDG devices.

Refer to the Experimental Section for additional details about the growth, transfer, and fabrication process. In Figure 1d i-iii, the optical images of the CVDG sample are presented. The representative SEM images of a suspended and a collapsed CVD graphene device are presented in Supporting Information Figure S1.

To characterize the crystallinity of the CVDG film, a customized TEM grid process was followed to suspend CVDG directly on copper foils (growth substrate). Scanning electron microscope (SEM) image of the custom TEM grid sample is presented in Supporting Information Figure S2. This allowed for the preparation of ultra-clean, polymer-free suspend CVDG films for TEM characterization. In Figure 1e,f, the intensity line profile and the selected area electron diffraction (SAED) image of the CVDG film are presented. The sixfold symmetry is observable in the SAED image, and the intensity profile provides insight into the single-layer nature of the CVDG film and the crystallinity of the imaged area of the CVDG film.^34,35^ The TEM image of the CVD graphene region wherein the SAED image was collected is presented in Supporting Information Figure S3. In Supporting Information Figure S3, the elemental analysis of the CVD graphene region was performed using energy-dispersive X-ray analysis (EDX). The presence of residual contamination from carbon, oxygen, and sulfur elements is observable in EDX characterization (see Figure S4 a–c) of the suspended graphene film region presented in Supporting Information Figure S4.

**Figure 2:**
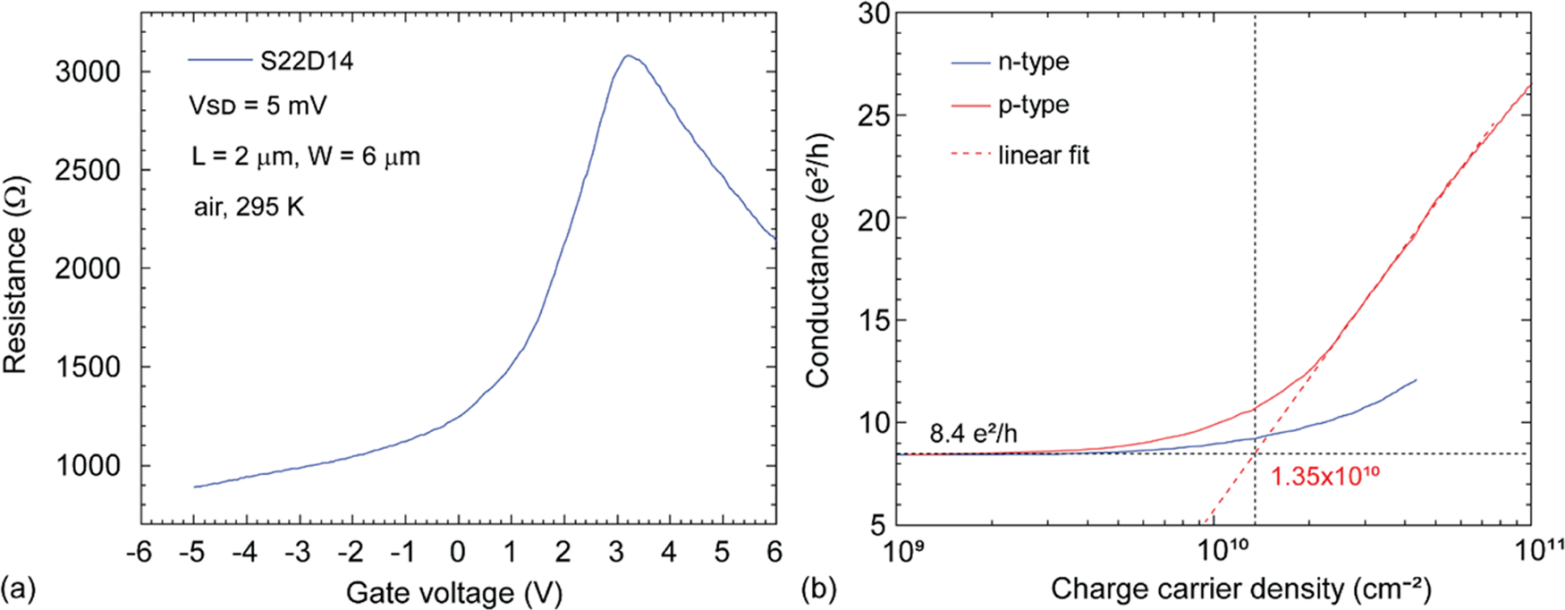
Field-effect characterization of the suspended CVDG devices. (a) Gate voltage (V) vs resistance (Ω) characteristic of CVDG device S22D14 (*L* = 2 μm, *W* = 6 μm) with *V*_SD_ = 5 mV. Note: All of the characterization was performed under standard cleanroom conditions (295 K, 45% RH) and ambient pressure. (b) Charge carrier density (cm^–2^) vs conductance (e^2^/h) of p-type (red curve) and n-type (blue curve) characteristics of S22D14. The linear fit to the p-type characteristic is highlighted using a red dotted line.

In Figure 2, field-effect characterization of the suspended CVDG device S22D14 (*L* = 2 μm, *W* = 6 μm) is presented. In Figure 2a, a shift in the charge neutrality point (CNP) ∼3.2 V due to hole doping is observable. Note that these measurements (*V*_SD_ = 5 mV) were performed in air, under ambient pressure, and in standard cleanroom conditions (45% RH, 295 K). Additional details related to the measurement acquisition are provided in the Experimental Section. In Figure 2b, the charge carrier density versus the conductance of S22D14 is presented.

The residual charge carrier density at the CNP for holes was extracted by considering the charge carrier density at which the slope of the p-type (hole) conductance (red dotted curve) intersects with the minimum conductance of ∼8.4 e^2^/h (horizontal black dotted line). The residual charge carrier density was estimated ∼at 1.35 × 10^10^ cm^–2^ by using a linear fit (red dotted curve, Figure 2b) to the p-type (red curve, Figure 2b) characteristic. The minimum conductance of the device is ∼8.4 e^2^/h. The hole mobility was estimated ∼at 24900 cm^2^/Vs, and the electron mobility at ∼7000 cm^2^/Vs. Additional details related to mobility extraction are presented in Supporting Information Figure S5.

**Figure 3:**
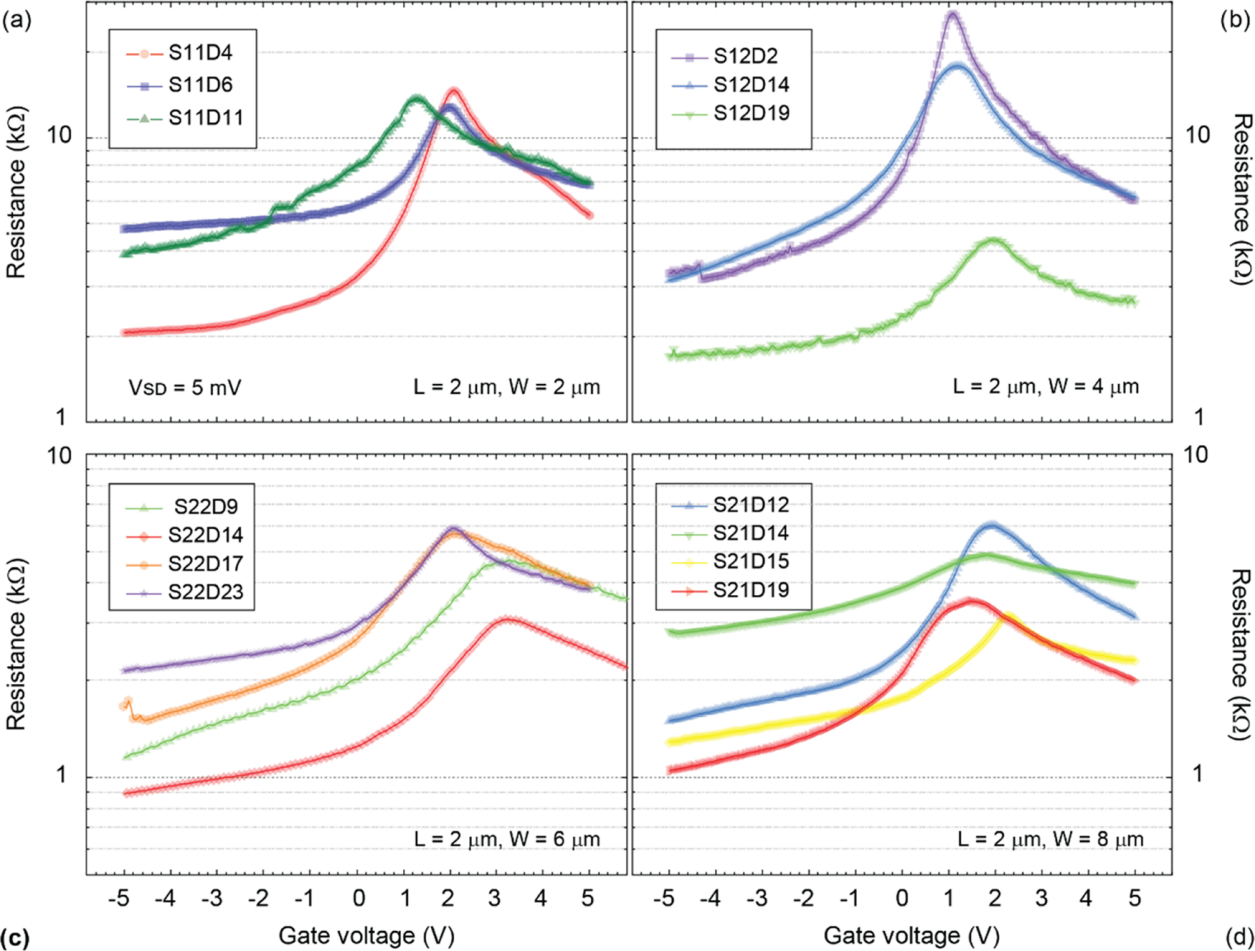
Field-effect characterization of the suspended CVDG devices with fixed length and varying width with *V*_SD_ = 5 mV. Gate voltage (V) vs resistance (kΩ) characteristics of (a) batch S11 (*L* = 2 μm, *W* = 2 μm, three samples), (b) batch S12 (*L* = 2 μm, *W* = 4 μm, three samples), (c) batch S22 (*L* = 2 μm, *W* = 6 μm, four samples), and (d) batch S21 (*L* = 2 μm, *W* = 8 μm, four samples). Note: All of the characterization was performed under standard cleanroom conditions (295 K, 45% RH) and ambient pressure.

To study the influence of the device aspect ratio (L/W), CVDG devices with fixed lengths (*L* = 2 μm) and varying widths were fabricated. The width of the devices was varied from *W* = 2 μm (S11) to 4 μm (S12), 6 μm (S22), and 8 μm (S21). The field-effect characterization of the different sample types is presented in Figure 3 a–d.

**Figure 4:**
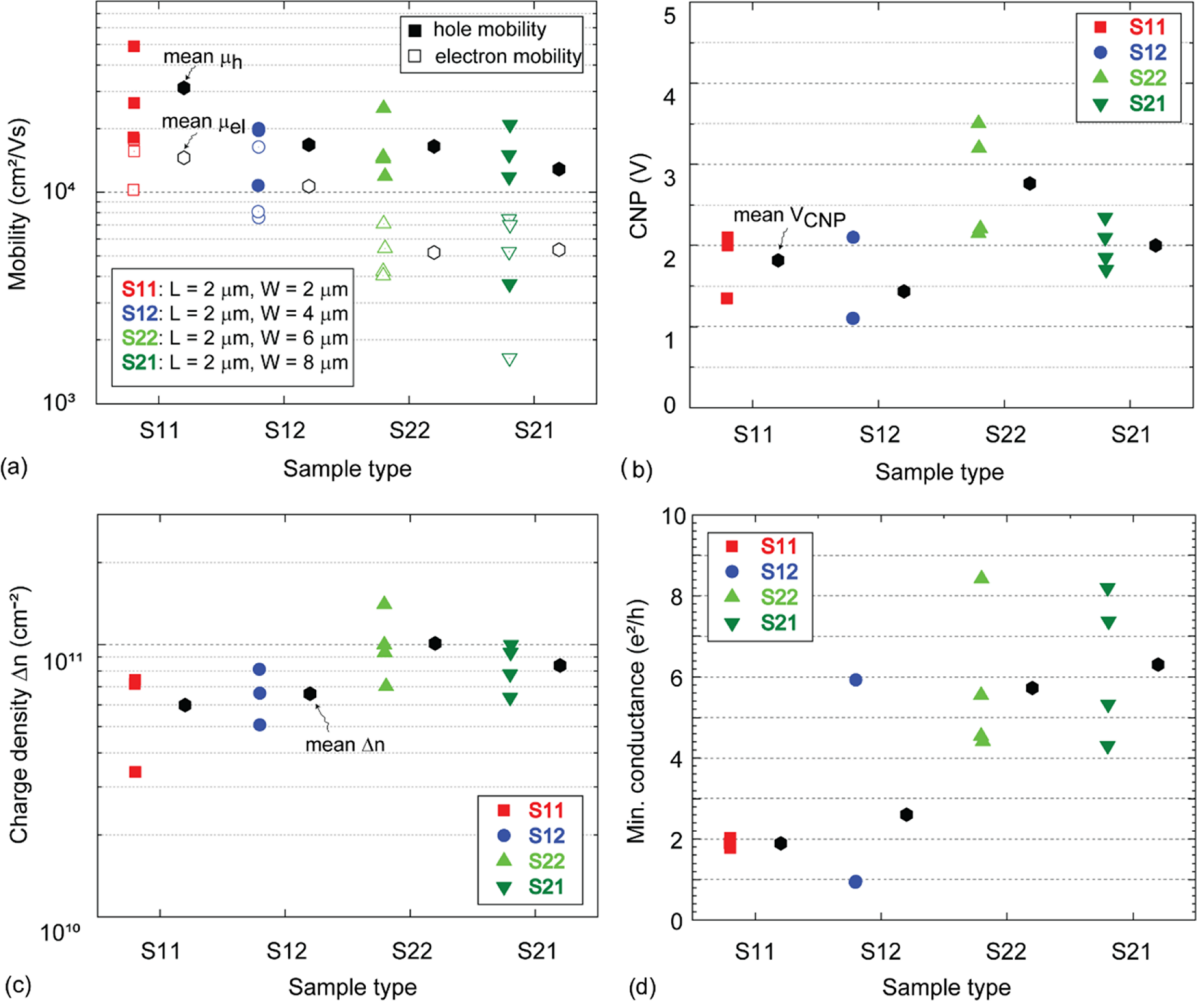
(a) Sample type vs field-effect mobility (cm^2^/Vs) characterization of the suspended CVDG devices with hole mobility (filled data points) and electron mobility (unfilled data points). The extracted hole and electron mobility of S11 (red squares, three devices), S12 (blue circles, three devices), S22 (light green triangle, four devices), and S21 (dark green inverted triangle, four devices) are grouped according to their sample type. Sample-dependent average mobility of holes (hexagon, filled) and electrons (hexagon, unfilled) as highlighted next to the data points. (b) Sample type vs CNP (V), (c) sample type vs FWHM Δ*n* (cm^–2^), and (d) sample type vs minimum conductance (e^2^/h), with the sample-dependent average (hexagon, filled) highlighted next to the data points.

The CNP of all devices is within the measurement window ± 5 V gate voltage and > 0 V in all of the devices, indicating the presence of p-doping. In Figure 4, the field-effect mobility of holes (μ_h_) and electrons (μ_el_), CNP, and the full width at half-maximum (FWHM) in charge carrier density (Δ*n*) of all devices is presented. By considering a Gaussian peak fit to the field-effect characteristics, the FWHM in gate voltage and, thereby, in charge carrier density (Δ*n*) is estimated.

The average μ_h_ (μ_el_) in sample type S11 is ∼31000 cm^2^/Vs (∼14500 cm^2^/Vs), μ_h_ (μ_el_) in S12 is ∼16700 cm^2^/Vs (∼10600 cm^2^/Vs), μ_h_ (μ_el_) in S22 is ∼16400 cm^2^/Vs (∼5100 cm^2^/Vs), and μ_h_ (μ_el_) in S21 is ∼12800 cm^2^/Vs (∼5300 cm^2^/Vs). The average μ_h_ (μ_el_) across all of the sample types is >19000 cm^2^/Vs (> 8900 cm_2_/Vs), see: Figure 4a. A gradual decrease in μ_h_ and μ_el_ with the increase in sample width is observable. The hole mobility μ_h_ is higher than the electron mobility μ_el_ in all of the samples. The μ_h_/μ_el_ ratio for each of the sample types is S11 ∼2.1, S12 ∼1.5, S22 ∼3.2, and S21 ∼2.4, with the overall average μ_h_/μ_el_ across all sample types being ∼2.3. Several factors, such as electrostatic potential fluctuations at the contact regions, strain, charged impurities, and electron–phonon scattering, can introduce such an asymmetry in field-effect characteristics.^36,37^ Studies to selectively suppress hole and electron conductance via molecular doping (charged impurities) have been reported.^37^

A minor shift in the CNP is observable in all of the devices (see: Figure 3). In Figure 4b, the CNP of all of the devices is presented, with an average CNP across all of the devices ∼ + 2 V. This indicates the presence of p-type doping in these devices. However, a slight increase in the CNP is observable with the increase in sample width (see Figure 4b). The average doping density n_0_ across all samples is ∼3.2 × 10^10^ cm^–2^. P-type doping of the samples can occur during fabrication and on exposure to photoresist and ambient measurement conditions. Residual p-type doping correlates well with the observation of a larger hole conductance compared to the electron conductance in Figure 2b. In addition, the FWHM (Δ*n*) parameter from the field-effect characteristics can be used to study the impact on mobility.^32^

A model with an inverse power law dependence between mobility and Δ*n* has been reported by considering impurity scattering as a primary factor influencing sample mobility.^32^ In Figure 4c, the sample-dependent average Δ*n* is increasing, S11_Δ*n*_ ∼ 6 × 10^10^ cm^–2^, S12_Δ*n*_ ∼ 6.6 × 10^10^ cm^–2^, S22_Δ*n*_ ∼ 1 × 10^11^ cm^–2^, and S21_Δ*n*_ ∼ 8.4 × 10^10^ cm^–2^. In Figure 4d, the extracted minimum conductance values of all devices are presented. In both Figure 4c,d, an increasing trend that correlates with the decreasing sample-dependent average mobility (see: Figure 4a) is observable. The Δ*n* increases by an average ∼1.9 × 10^10^ cm^–2^ with the increase in sample width. The increase in Δ*n* and minimum conductance values and the decreasing mobility can be collectively attributed to the increase in impurity or defect density (*n*_imp_) in these samples.^31,32^ Considering the exposure to polymeric contamination during the fabrication process, current annealing characterization was performed before field-effect characterization. Current annealing is a useful technique for removing residues on suspended graphene samples.^25,27,29,38,39^ The influence of current annealing on CVD graphene characteristics is presented in Supporting Information S6. Other groups have reported current density >10^**8**^A/cm^**2**^ during the current annealing process at low temperatures and under high-vacuum conditions.^25,38^

In Supporting Information Figure S7, the current annealing characteristics of S11D4 are presented. Note that in our study, the current annealing was performed in air and at room temperature, which is a typical operating environment for several sensing applications. A current density of ∼0.43 × 10^8^ A/cm^**2**^ was observable in S11D4 (*L* = 2 μm, *W* = 2 μm) at a *V*_**SD**_ ∼1V (see: Figure S7). In Supporting Information Figures S8S–11, the current annealing characteristics of additional device types are presented. The current density (at *V*_SD_ ∼1V) is ∼0.43 × 10^8^ A/cm^**2**^ in S11D4, ∼0.12 × 10^8^ A/cm^**2**^ in S12D14, 0.18 × 10 ^8^A/cm^**2**^ in S22D21, and ∼0.11 × 10^8^ A/cm^**2**^ in S21D19. Due to the inverse relation between the current density and sample width, a decreasing current density trend with the increase in sample width is observable at a fixed *V*_**SD**_ ∼ 1V.

The reported current density in high-mobility suspended graphene devices by other groups is >10^8^ A/cm^2^, which is at least an order of magnitude larger than the current density values observed in our devices. Note that the breakdown temperature (*T*_BD_) of graphene samples in the air (*T*_BD, air_ ∼ 873 K) is significantly lower in comparison to *T*_BD_ in vacuum (*T*_BD, vacuum_ ∼ 1420 to 2860 K).^40^ In our case, the measurements in higher current density regimes with *V*_SD_ > 2V were limited to minimize the potential risk of device degradation from thermal oxidation in air. The gradual decrease in the field-effect mobility with the increasing width can be explained by the decreasing current density trend across the CVDG device types, i.e., higher residual charge impurities remain on the samples due to the limited current annealing of larger CVDG devices. EDX characterization of suspended CVD graphene on custom grids was performed to characterize the elemental residues on CVD graphene. In Supporting Information Figure S4, the elemental presence of carbon, oxygen, and sulfur was identified on the suspended CVD graphene films. The presence of such residual elemental composition can be attributed to processing-related conditions. Considering the presence of oxygen and sulfur atoms in the copper etchant (ammonium persulfate), residues from the copper etching process support the presence of residual oxygen and sulfur groups on the CVD graphene surface.

In Table 1, a brief comparison of field-effect mobility at room temperature is highlighted for type 1 (on-substrate), type 2 (encapsulated), and type 3 (suspended) graphene devices. We can notice the higher field-effect mobility in suspended and encapsulated CVDG devices compared to CVDG devices fabricated directly on the substrate. Considering the unavoidable exposure to organic polymers during the fabrication of type 3 graphene devices, the current annealing technique effectively removes organic residues from the graphene surface. However, the current annealing conditions can influence the device performance significantly.

**Table 1:** Graphene Mobility Comparison of Type 1, Type 2, and Type 3 Devices at Room Temperature

device type & substrate		mobility (cm^2^/Vs)	measurement condition	graphene type
type 1, On SiO_2_/Si	ref (11)	∼8600	air	CVD
	ref (41)	∼9104	air	CVD
	ref (42)	∼12 000	vacuum	CVD
	ref (16)	∼16 000	d	CVD
	ref (43)	∼18 000	vacuum	CVD
type 2, encapsulated^a^	ref (44)	∼70 000	d	CVD
	ref (20)	∼76 000^b^	d	CVD
	ref (45)	∼135 000	vacuum	CVD
	ref (24)	∼140 000	vacuum	exfoliated
type 3, suspended	ref (46)	∼20 000	d	exfoliated
	ref (47)	∼60 000	liquid (anisole)	exfoliated
	ref (28)	∼70 000	vacuum	exfoliated
	ref (48)	∼120 000^c^	vacuum	exfoliated
	this work (S11D4)	∼48 000	air	CVD

a Encapsulated with exfoliated hBN flakes.

b Data manually extracted.

c Measured at 240 K.

d Condition unknown.

Note that the current annealing conditions vary considerably between S11D4 and the exfoliated graphene-based type 3 devices in Table 1. In type 3 exfoliated devices, the current annealing process was carried out under low vacuum and low-temperature conditions. This helps minimize the thermal oxidation of graphene films during the annealing process. However, in the case of S11D4 and the other reported CVDG devices in this report, the current annealing was performed in air and at room temperature. Performing current annealing of CVDG devices under low vacuum conditions is favorable to further improve the field-effect mobility metrics at room temperature.

## Conclusion

In summary, we presented a fabrication approach to realize suspended CVD graphene devices with high hole field-effect mobility of up to ∼4.8 × 10^4^ cm^2^/Vs at room temperature and ambient pressure using standard cleanroom processing methods. A width-dependent study of the suspended CVD graphene devices showed an increase in the FWHM of the field-effect characteristics and a decrease in mobility. This observation is attributed to the increasing charge impurity scattering in larger suspended CVD graphene devices. Further processing conditions such as thermal annealing, contact passivation, and measurement under inert conditions can be beneficial in further improving the performance metrics of the suspended CVDG devices and uniformly lowering the impurity density across the samples. The demonstration of high-field-effect mobility in suspended CVD graphene devices operating in ambient conditions strengthens the prospects of developing scalable, high-performance sensing solutions with synthetic graphene films.

## Experimental Section

### CVD Graphene Growth

CVD synthesis of graphene was performed using a low-pressure, hot-wall CVD reactor from Graphene Square, Inc. The copper foils were first thermally annealed at 1030 °C for four hours. The CVD synthesis of graphene was performed at 1000 °C using a methane/hydrogen ratio of ∼ 1:13. After a growth time of ∼45 min, the chamber was cooled to room temperature in argon and hydrogen gas flow.

### Graphene Transfer

A PMMA 50K layer was spin-coated (4000 RPM, 40 s) on graphene grown on the top side of copper foil. The graphene on the bottom side of the copper foil was removed in oxygen plasma (30 W, 45 s, 30/15 sccm of oxygen/argon). Ammonium persulfate solution (50 mM) was used to etch the copper layer. After the etching process, the PMMA/CVDG layer was transferred to the DIW solution. The PMMA/CVDG layer was then picked up using the target substrate and let to dry. The PMMA layer was dissolved in acetone and rinsed in IPA.

### Graphene Patterning

Two-terminal etch mask pattern was realized using the AZ1512 (4000 RPM, 40 s) photolithography process. The exposed graphene layer was removed using an oxygen plasma process (30 W, 45 s, 30/15 sccm of oxygen/argon). After graphene patterning, the etch mask was removed in acetone and rinsed in IPA.

### Graphene Suspension

An etch mask pattern for graphene suspension was realized using a second photolithography step using AZ1512 (4000 RPM, 40 s) photolithography process. Buffered oxide etching (BOE) step was performed (∼15 min) to remove the SiO_2_. After the BOE process, the sample was rinsed in DIW solution (∼15 min). The DIW rinse step was repeated 3x and then moved to IPA. Next, the etch mask is removed in acetone (∼15 min) and then moved to IPA. To prevent stiction-related challenges, a Tousimis critical point dryer was used for the suspension process. In total, 17 active devices were characterized during this study with a fabrication yield of ∼17%.

### Electrical Characterization

The field-effect characterization of the devices was performed in air and under ambient cleanroom conditions (295 K, 45%RH) using a wafer probe station (Cascade Summit 12000B-AP) equipped with an Agilent B1500A semiconductor parameter analyzer.

### TEM Characterization

Selected area electron diffraction, scanning transmission electron microscopy (STEM), and energy-dispersive X-ray analysis (EDX) were carried out on a TFS Talos F200X running at an 80 kV acceleration voltage.

## Supporting Information Available

Representative SEM images of suspended and collapsed CVD graphene device (S1); SEM (S2) and TEM (S3) images of custom TEM grid with the suspended CVD graphene; EDX characterization of the suspended CVD graphene films (S4); field-effect mobility estimation (S5); and current annealing measurements of suspended CVD devices (S6–S11) (PDF)

## References

[ref1] Castro Neto A. H.; Guinea F.; Peres N. M. R.; Novoselov K. S.; Geim A. K. The Electronic Properties of Graphene. Rev. Mod. Phys. 2009, 81, 109 10.1103/RevModPhys.81.109.

[ref2] Das Sarma S.; Adam A.; Hwang E. H.; Rossi E. Electronic Transport in Two-Dimensional Graphene. Rev. Mod. Phys. 2011, 83, 407 10.1103/RevModPhys.83.407.

[ref3] Auton G.; Zhang J.; Kumar R. K.; Wang H.; Zhang X.; Wang Q.; Hill E.; Song A. Graphene Ballistic Nano-Rectifier with Very High Responsivity. Nat. Commun. 2016, 7, 11670 10.1038/ncomms11670. 27241162 PMC4895026

[ref4] Banszerus L.; Schmitz M.; Engels S.; Goldsche M.; Watanabe K.; Taniguchi T.; Beschoten B.; Stampfer C. Ballistic Transport Exceeding 28 μm in CVD Grown Graphene. Nano Lett. 2016, 16, 1387–1391. 10.1021/acs.nanolett.5b04840. 26761190

[ref5] Wilmart Q.; Berrada S.; Torrin D.; Nguyen V. H.; Fève G.; Berroir J-M.; Dollfus P.; Plaçais B. A Klein-Tunneling Transistor with Ballistic Graphene. 2D Mater. 2014, 1, 11006 10.1088/2053-1583/1/1/011006.

[ref6] Schmidt F. E.; Jenkins M. D.; Watanabe K.; Taniguchi T.; Steele G. A. A Ballistic Graphene Superconducting Microwave Circuit. Nat. Commun. 2018, 9, 4069 10.1038/s41467-018-06595-2. 30287816 PMC6172216

[ref7] Chen S.; Han Z.; Elahi M. M.; Habib K. M. M.; Wang L.; Wen B.; Gao Y.; Taniguchi T.; Watanabe K.; Hone J.; Ghosh A. W.; Dean C. R. Electron Optics with p-n Junctions in Ballistic Graphene. Science 2016, 353, 1522–1525. 10.1126/science.aaf5481. 27708099

[ref8] Ribeiro-Palau R.; Lafont F.; Brun-Picard J.; Kazazis D.; Michon A.; Cheynis F.; Couturaud O.; Consejo C.; Jouault B.; Poirier W.; Schopfer F. Quantum Hall Resistance Standard in Graphene Devices under Relaxed Experimental Conditions. Nat. Nanotechnol. 2015, 10, 965–971. 10.1038/nnano.2015.192. 26344181

[ref9] Tzalenchuk A.; Lara-Avila S.; Kalaboukhov A.; Paolillo S.; Syväjärvi M.; Yakimova R.; Kazakova O.; Janssen T. J. B. M.; Fal’ko V.; Kubatkin S. Towards a Quantum Resistance Standard based on Epitaxial Graphene. Nat. Nanotechnol. 2010, 5, 186–189. 10.1038/nnano.2009.474. 20081845

[ref10] Bae S.; Kim H.; Lee Y.; Xu X.; Park J.-S.; Zheng Y.; Balakrishnan J.; Lei T.; Ri Kim H.; Song Y. I.; Kim Y.-J.; Kim K. S.; Özyilmaz B.; Ahn J.-H.; Hong B. H.; Iijima S. Roll-to-Roll Production of 30-inch Graphene Films for Transparent Electrodes. Nat. Nanotechnol. 2010, 5, 574–578. 10.1038/nnano.2010.132. 20562870

[ref11] Li J.; Chen M.; Samad A.; Dong H.; Ray A.; Zhang J.; Jiang X.; Schwingenschlögl U.; Domke J.; Chen C.; Han Y.; Fritz T.; Ruoff R. S.; Tian B.; Zhang X. Wafer-Scale Single-Crystal Monolayer Graphene Grown on Sapphire Substrate. Nat. Mater. 2022, 21, 740–747. 10.1038/s41563-021-01174-1. 35058609

[ref12] Li X.; Zhu Y.; Cai W.; Borysiak M.; Han B.; Chen D.; Piner R. D.; Colombo L.; Ruoff R. S. Transfer of Large-Area Graphene Films for High-Performance Transparent Conductive Electrodes. Nano Lett. 2009, 9, 4359–4363. 10.1021/nl902623y. 19845330

[ref13] Reina A.; Jia X.; Ho J.; Nezich D.; Son H.; Bulovic V.; Dresselhaus M. S.; Kong J. Large Area, Few-Layer Graphene Films on Arbitrary Substrates by Chemical Vapor Deposition. Nano Lett. 2009, 9, 30–35. 10.1021/nl801827v. 19046078

[ref14] Zhang J.; Lin L.; Jia K.; Sun L.; Peng H.; Liu Z. Controlled Growth of Single-Crystal Graphene Films. Adv. Mater. 2020, 32, 1903266 10.1002/adma.201903266. 31583792

[ref15] Hao Y.; Bharathi M. S.; Wang L.; Liu Y.; Chen H.; Nie S.; Wang X.; Chou H.; Tan C.; Fallahazad B.; Ramanarayan H.; Magnuson C. W.; Tutuc E.; Yakobson B. I.; McCarty K. F.; Zhang Y.-W.; Kim P.; Hone J.; Colombo L.; Ruoff R. S. The Role of Surface Oxygen in the Growth of Large Single-Crystal Graphene on Copper. Science 2013, 342, 720–723. 10.1126/science.1243879. 24158906

[ref16] Yan Z.; Peng Z.; Tour J. M. Chemical Vapor Deposition of Graphene Single Crystals. Acc. Chem. Res. 2014, 47, 1327–1337. 10.1021/ar4003043. 24527957

[ref17] Thodkar K.; Abbassi M. E.; Lüönd F.; Overney F.; Schönenberger C.; Jeanneret B.; Calame M. Comparative Study of Single and Multi Domain CVD Graphene using Large-Area Raman Mapping and Electrical Transport Characterization. Phys. Status Solidi RRL 2016, 10, 807–811. 10.1002/pssr.201600211.

[ref18] Yu Q.; Lian J.; Siriponglert S.; Li H.; Chen Y. P.; Pei S-S.. Graphene Segregated on Ni Surfaces and Transferred to Insulators. Appl. Phys. Lett. 2008, 93, 113103 10.1063/1.2982585.

[ref19] Wang Y.; Zheng Y.; Xu X.; Dubuisson E.; Bao Q.; Lu J.; Loh K. P. Electrochemical Delamination of CVD-Grown Graphene Film: Toward the Recyclable Use of Copper Catalyst. ACS Nano 2011, 5, 9927–9933. 10.1021/nn203700w. 22034835

[ref20] Banszerus L.; Schmitz M.; Engels S.; Dauber J.; Oellers M.; Haupt F.; Watanabe K.; Taniguchi T.; Beschoten B.; Stampfer C. Ultrahigh-Mobility Graphene Devices from Chemical Vapor Deposition on Reusable Copper. Sci. Adv. 2015, 1, e1500222 10.1126/sciadv.1500222. 26601221 PMC4646786

[ref21] Lock E. H.; Baraket M.; Laskoski M.; Mulvaney S. P.; Lee W. K.; Sheehan P. E.; Hines D. R.; Robinson J. T.; Tosado J.; Fuhrer M. S.; Hernández S. C.; Walton S. G. High-Quality Uniform Dry Transfer of Graphene to Polymers. Nano Lett. 2012, 12, 102–107. 10.1021/nl203058s. 22128775

[ref22] Chen J.-H.; Jang C.; Xiao S.; Ishigami M.; Fuhrer M. S. Intrinsic and Extrinsic Performance Limits of Graphene Devices on SiO2. Nat. Nanotechnol. 2008, 3, 206–209. 10.1038/nnano.2008.58. 18654504

[ref23] Dean C. R.; Young A. F.; Meric I.; Lee C.; Wang L.; Sorgenfrei S.; Watanabe K.; Taniguchi T.; Kim P.; Shepard K. L.; Hone J. Boron Nitride Substrates for High-Quality Graphene Electronics. Nat. Nanotechnol. 2010, 5, 722–726. 10.1038/nnano.2010.172. 20729834

[ref24] Wang L.; Meric I.; Huang P. Y.; Gao Q.; Gao Y.; Tran H.; Taniguchi T.; Watanabe K.; Campos L. M.; Muller D. A.; Guo J.; Kim P.; Hone J.; Shepard K. L.; Dean C. R. One-Dimensional Electrical Contact to a Two-Dimensional Material. Science 2013, 342, 614–617. 10.1126/science.1244358. 24179223

[ref25] Bolotin K. I.; Sikes K. J.; Jiang Z.; Klima M.; Fudenberg G.; Hone J.; Kim P.; Stormer H. L. Ultrahigh Electron Mobility in Suspended Graphene. Solid State Commun. 2008, 146, 351–355. 10.1016/j.ssc.2008.02.024.

[ref26] Du X.; Skachko I.; Barker A.; Andrei E. Y. Approaching Ballistic Transport in Suspended Graphene. Nat. Nanotechnol. 2008, 3, 491–495. 10.1038/nnano.2008.199. 18685637

[ref27] Rickhaus P.; Maurand R.; Liu M.-H.; Weiss M.; Richter K.; Schönenberger C. Ballistic Interferences in Suspended Graphene. Nat. Commun. 2013, 4, 2342 10.1038/ncomms3342. 23946010

[ref28] Tombros N.; Veligura A.; Junesch J.; van den Berg J. J.; Zomer P. J.; Wojtaszek M.; Marun I. J. V.; Jonkman H. T.; van Wees B. J. Large Yield Production of High Mobility Freely Suspended Graphene Electronic Devices on a Polydimethylglutarimide based Organic Polymer. J. Appl. Phys. 2011, 109, 93702 10.1063/1.3579997.

[ref29] Grushina A. L.; Ki D. K.; Morpurgo A. F. A Ballistic pn Junction in Suspended Graphene with Split Bottom Gates. Appl. Phys. Lett. 2013, 102, 223102 10.1063/1.4807888.

[ref30] Adam S.; Hwang E. H.; Galitski V. M.; Das Sarma S. A Self-Consistent Theory for Graphene Transport. Proc. Natl. Acad. Sci. U.S.A. 2007, 104, 18392–18397. 10.1073/pnas.0704772104. 18003926 PMC2141788

[ref31] Chen J.-H.; Jang C.; Adam S.; Fuhrer M. S.; Williams E. D.; Ishigami M. Charged-Impurity Scattering in Graphene. Nat. Phys. 2008, 4, 377–381. 10.1038/nphys935.

[ref32] Gosling J. H.; Makarovsky O.; Wang F.; Cottam N. D.; Greenaway M. T.; Patanè A.; Wildman R. D.; Tuck C. J.; Turyanska L.; Fromhold T. M. Universal Mobility Characteristics of Graphene Originating from Charge Scattering by Ionised Impurities. Commun. Phys. 2021, 4, 30 10.1038/s42005-021-00518-2.

[ref33] Burson K. M.; Cullen W. G.; Adam S.; Dean C. R.; Watanabe K.; Taniguchi T.; Kim P.; Fuhrer M. S. Direct Imaging of Charged Impurity Density in Common Graphene Substrates. Nano Lett. 2013, 13, 3576–3580. 10.1021/nl4012529. 23879288

[ref34] Hernandez Y.; Nicolosi V.; Lotya M.; Blighe F. M.; Sun Z.; De S.; McGovern I. T.; Holland B.; Byrne M.; Gun’Ko Y. K.; Boland J. J.; Niraj P.; Duesberg G.; Krishnamurthy S.; Goodhue R.; Hutchison J.; Scardaci V.; Ferrari A. C.; Coleman J. N. High-Yield Production of Graphene by Liquid-Phase Exfoliation of Graphite. Nat. Nanotechnol. 2008, 3, 563–568. 10.1038/nnano.2008.215. 18772919

[ref35] Meyer J. C.; Geim A. K.; Katsnelson M. I.; Novoselov K. S.; Obergfell D.; Roth S.; Girit C.; Zettl A. On the Roughness of Single- and Bi-layer Graphene Membranes. Solid State Commun. 2007, 143, 101–109. 10.1016/j.ssc.2007.02.047.

[ref36] Srivastava P. K.; Arya S.; Kumar S.; Ghosh S. Relativistic nature of carriers: Origin of Electron-Hole Conduction Asymmetry in Monolayer Graphene. Phys. Rev. B 2017, 96, 241407 10.1103/PhysRevB.96.241407.

[ref37] Farmer D. B.; Golizadeh-Mojarad R.; Perebeinos V.; Lin Y.-M.; Tulevski G. S.; Tsang J. C.; Avouris P. Chemical Doping and Electron–Hole Conduction Asymmetry in Graphene Devices. Nano Lett. 2009, 9, 388–392. 10.1021/nl803214a. 19102701

[ref38] Moser J.; Barreiro A.; Bachtold A. Current-Induced Cleaning of Graphene. Appl. Phys. Lett. 2007, 91, 163513 10.1063/1.2789673.

[ref39] Nam Y. Current Annealing Behavior in Suspended Graphene. J. Korean Phys. Soc. 2021, 79, 76–80. 10.1007/s40042-021-00221-z.

[ref40] Dorgan V. E.; Behnam A.; Conley H. J.; Bolotin K. I.; Pop E. High-Field Electrical and Thermal Transport in Suspended Graphene. Nano Lett. 2013, 13, 4581–4586. 10.1021/nl400197w. 23387323

[ref41] Tyagi A.; Mišeikis V.; Martini L.; Forti S.; Mishra N.; Gebeyehu Z. M.; Giambra M. A.; Zribi J.; Frégnaux M.; Aureau D.; Romagnoli M.; Beltram F.; Coletti C. Ultra-Clean High-Mobility Graphene on Technologically Relevant Substrates. Nanoscale 2022, 14, 2167–2176. 10.1039/D1NR05904A. 35080556

[ref42] Chen B.; Huang H.; Ma X.; Huang L.; Zhang Z.; Peng L. -M. How Good Can CVD-Grown Monolayer Graphene Be?. Nanoscale 2014, 6, 15255–15261. 10.1039/C4NR05664G. 25381813

[ref43] Lin L.; Zhang J.; Su H.; Li J.; Sun L.; Wang Z.; Xu F.; Liu C.; Lopatin S.; Zhu Y.; Jia K.; Chen S.; Rui D.; Sun J.; Xue R.; Gao P.; Kang N.; Han Y.; Xu H. Q.; Cao Y.; Novoselov K. S.; Tian Z.; Ren B.; Peng H.; Liu Z. Towards Super-Clean Graphene. Nat. Commun. 2019, 10, 1912 10.1038/s41467-019-09565-4. 31015405 PMC6478734

[ref44] De Fazio D.; Purdie D. G.; Ott A. K.; Braeuninger-Weimer P.; Khodkov T.; Goossens S.; Taniguchi T.; Watanabe K.; Livreri P.; Koppens F. H. L.; Hofmann S.; Goykhman I.; Ferrari A. C.; Lombardo A. High-Mobility, Wet-Transferred Graphene Grown by Chemical Vapor Deposition. ACS Nano 2019, 13, 8926–8935. 10.1021/acsnano.9b02621. 31322332

[ref45] Pezzini S.; Mišeikis V.; Pace S.; Rossella F.; Watanabe K.; Taniguchi T.; Coletti C. High-Quality Electrical Transport using Scalable CVD Graphene. 2D Mater. 2020, 7, 41003 10.1088/2053-1583/aba645.

[ref46] Bao W.; Liu G.; Zhao Z.; Zhang H.; Yan D.; Deshpande A.; LeRoy B.; Lau C. N. Lithography-Free Fabrication of High Quality Substrate-Supported and Freestanding Graphene Devices. Nano Res. 2010, 3, 98–102. 10.1007/s12274-010-1013-5.

[ref47] Newaz A. K. M.; Puzyrev Y. S.; Wang B.; Pantelides S. T.; Bolotin K. I. Probing Charge Scattering Mechanisms in Suspended Graphene by Varying its Dielectric Environment. Nat. Commun. 2012, 3, 734 10.1038/ncomms1740. 22415825

[ref48] Bolotin K. I.; Sikes K. J.; Hone J.; Stormer H. L.; Kim P. Temperature-Dependent Transport in Suspended Graphene. Phys. Rev. Lett. 2008, 101, 96802 10.1103/PhysRevLett.101.096802. 18851636

