# Supplementary material for: Enhanced Mobility in Suspended Chemical Vapor-Deposited Graphene Field-Effect Devices in Ambient Conditions
Source: ACS Appl Mater Interfaces. 2023 Jul 25;15(31):37756–63. doi: 10.1021/acsami.3c04012 (PMC10416145; doi:10.1021/acsami.3c04012)
Supplement: Supplementary file 1 — am3c04012_si_001.pdf [file am3c04012_si_001.pdf]

Supporting Information

Enhanced Mobility in Suspended Chemical Vapor-Deposited Graphene Field-Effect Devices in Ambient Conditions

Kishan Thodkar[1,*], *Fabian Gramm*[2]

[1]Micro- & Nanosystems, Department of Mechanical & Process Engineering, Tannenstrasse 3, ETH Zurich, 8052 Zurich, Switzerland

[2]ScopeM, Otto-Stern-Weg 3, ETH Zurich, 8093 Zurich, Switzerland

[*]email: kishant@ethz.ch

S1. SEM images of a suspended and a collapsed CVD graphene device

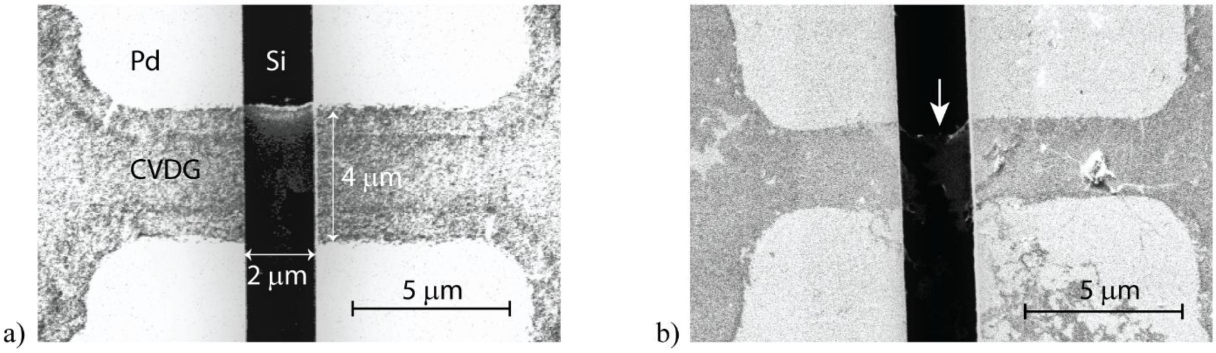

Figure S1. SEM images of **(a)** suspended CVD graphene device, and **(b)** CVD graphene collapsed on the back gate.

S2. SEM images of the custom TEM grid sample with suspended CVD graphene films

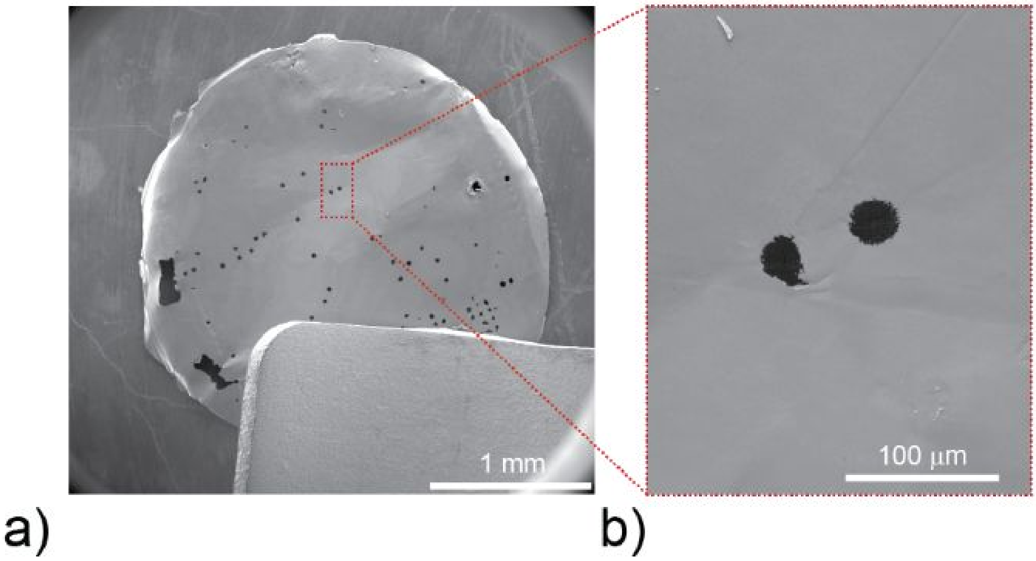

Figure S2. SEM characterization of **(a)** custom TEM grid sample with suspended CVD graphene films for TEM characterization. **(b)** Magnified regions of the custom TEM grid with suspended graphene regions are highlighted using a dotted red area.

S3. Transmission electron image of suspended CVD graphene

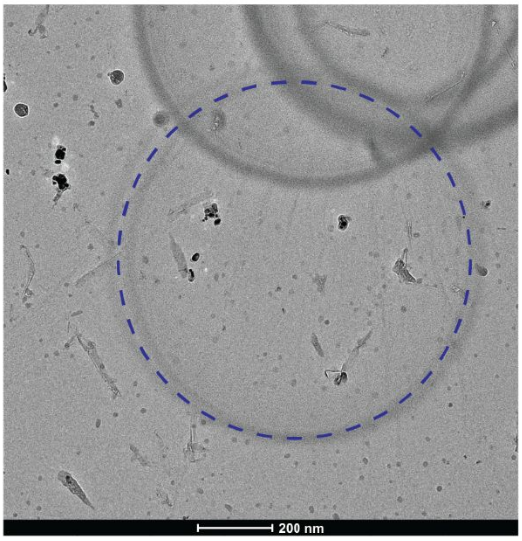

Figure S3. Transmission electron image of the CVD graphene area (blue dashed circle) for the collection of selected area electron diffraction image.

S4. Energy-dispersive X-ray (EDX) characterization of suspended CVD graphene

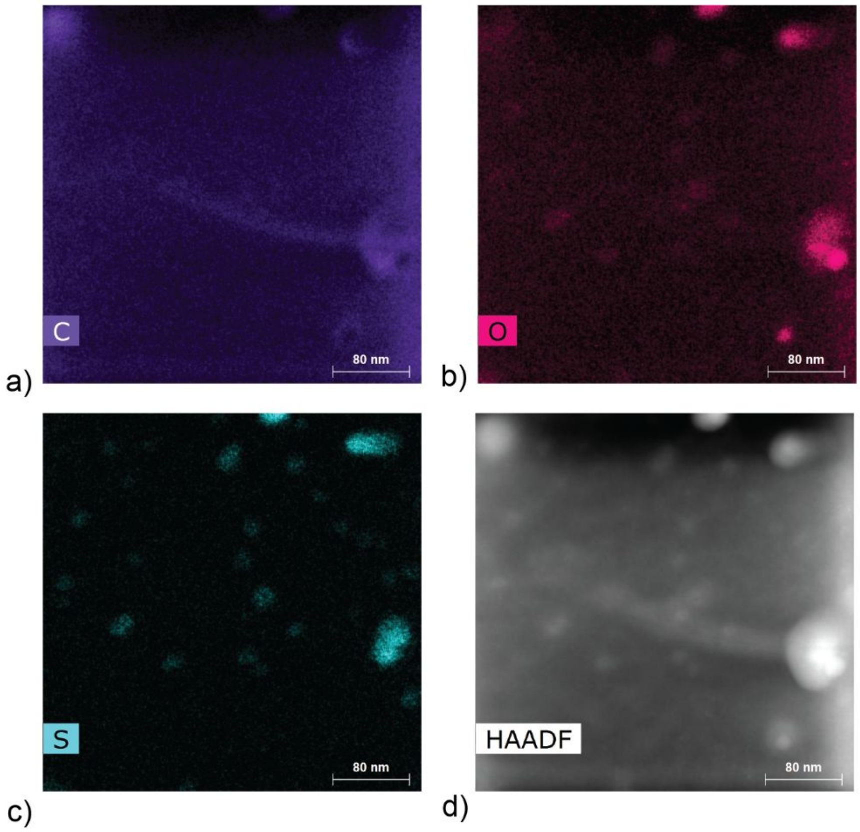

Figure S4. EDX characterization of suspended CVD graphene with elemental analysis depicting the presence of **(a)** Carbon, **(b)** Oxygen, **(c)** Sulphur, and **(d)** high-angle annular dark field (HAADF) image of the CVD graphene region (scale: 80 nm).

S5. Field-effect mobility extraction example of a suspended CVD graphene device

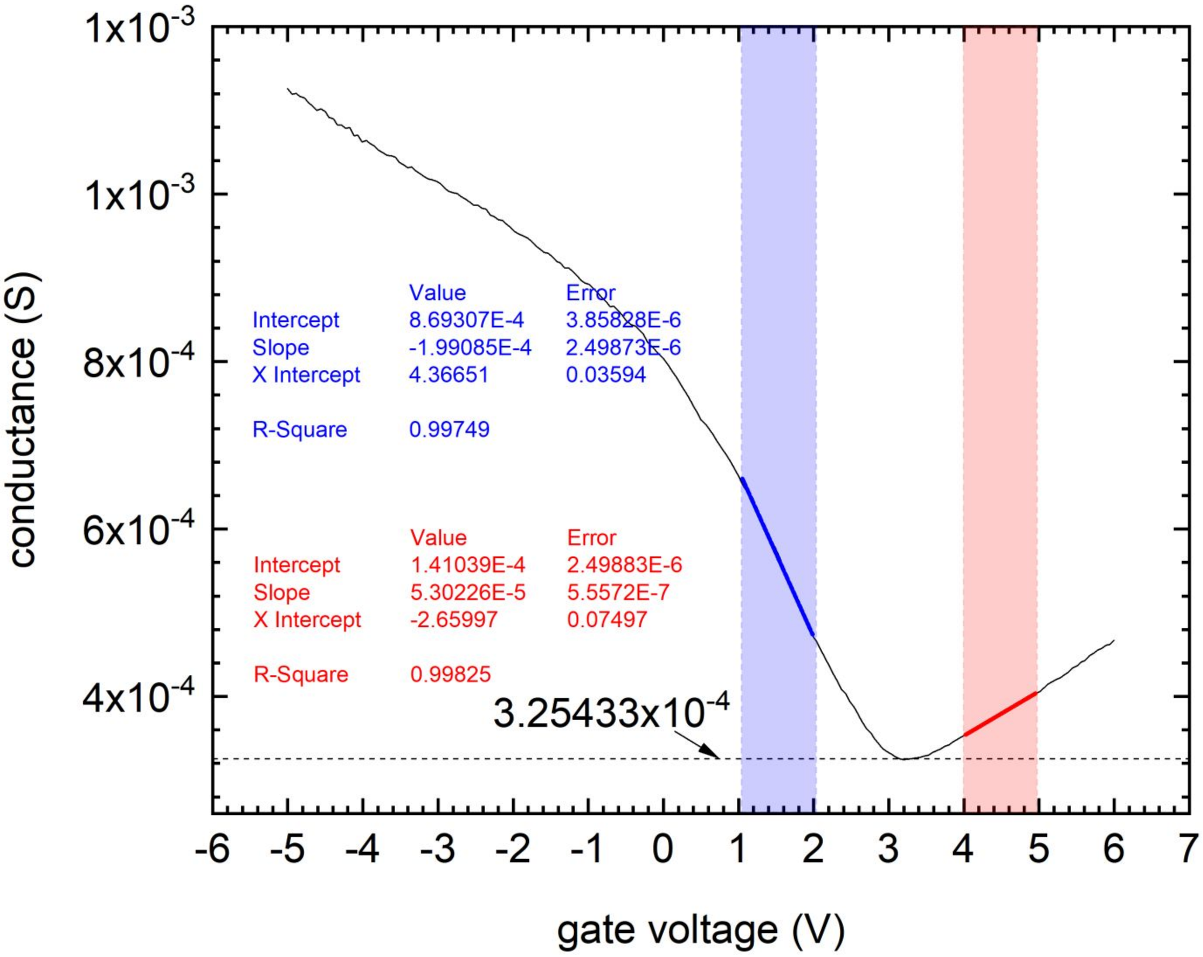

Figure S5. Field-effect mobility (cm^2/Vs) was extracted using $\mu = \frac{1}{Cgate}\frac{dG}{dVg}\frac{L}{W}$. The linear fit characteristics ($\frac{dG}{dVg}$) to the conductance (S) vs gate voltage (V) of S22D14 are highlighted in the blue and red lines near the CNP. The width (~1V) of the slope region is highlighted within the blue (holes) and red (electrons) regions. The minimum conductance value is highlighted using the dotted horizontal line. The gate capacitance ($Cgate$) ~24.9 aF/μm^2 (where ε_r = 1, distance to gate 'd' = 355 nm) and the device aspect ratio L/W = 0.334 for S22 samples. Note that given the suspended form of the graphene film, changes in the suspension height can occur with the change in gate voltage and contribute to the error in field-effect mobility estimate. For example, a suspension height decrease of ~10 nm can lead to an increase in Cg ~ 25.7 aF/μm^2.

S6. Influence of current annealing on CVD graphene devices

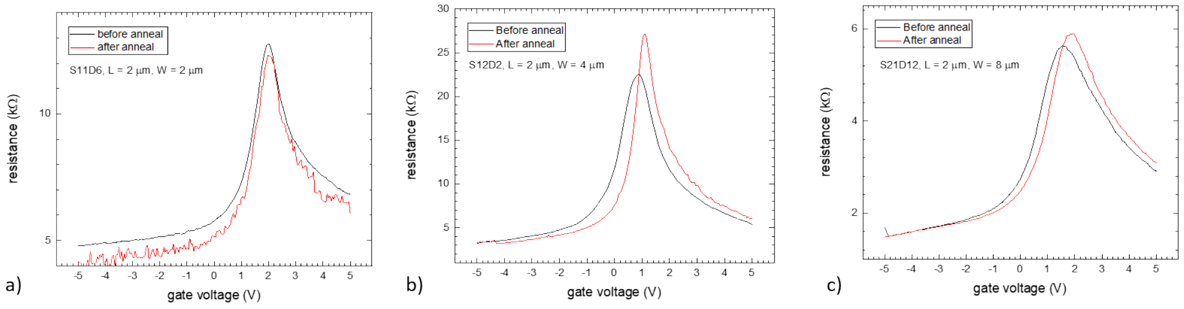

Figure S6. Field-effect characteristics comparison before and after the current annealing in devices **(a)** S11D6, **(b)** S12D2, and **(c)** S21D12.

S7. Current annealing measurements of CVD graphene device S11D4

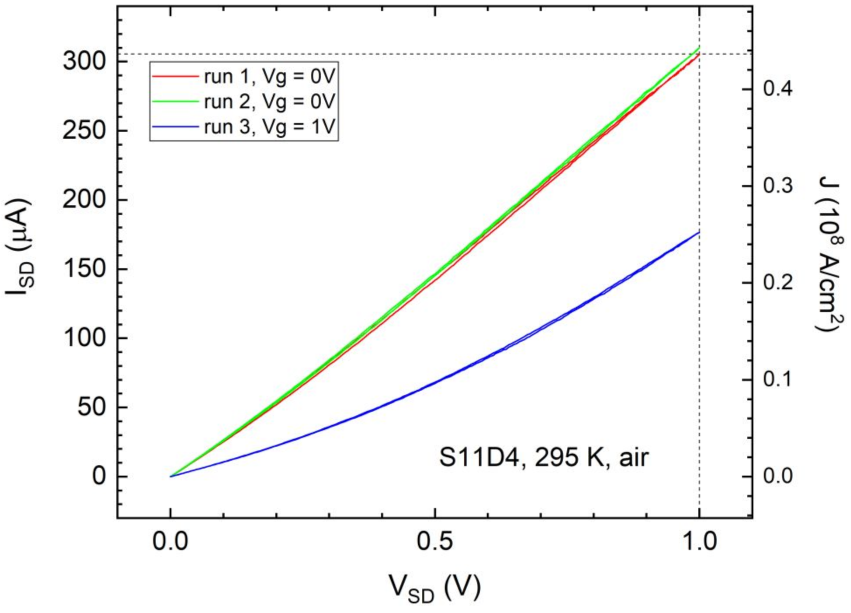

Figure S7. Current annealing characterization of CVDG device S11D4 (L = 2μm, W = 2μm) performed at 295K in air. Note the sample width W = 2 μm and single-layer graphene thickness of 0.35 nm were considered to estimate the current density J.

S8. Multiple current annealing measurements of CVD graphene device S11D2

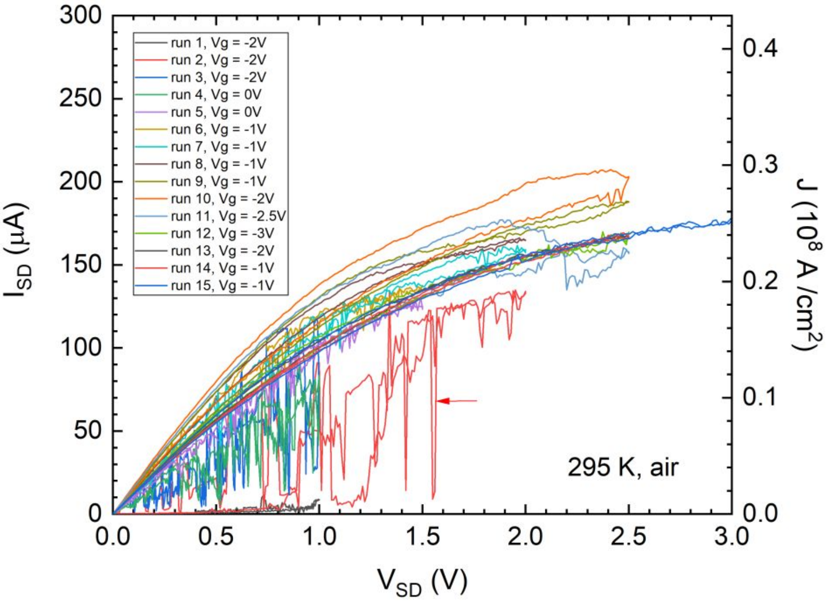

Figure S8. Current annealing characterization of CVDG device S11D2 (L = 2μm, W = 2μm) performed at 295K in air. Note the increase in current in run 2 (red line) and the subsequent current saturation at higher V_{SD}. FET characteristics could not be recorded after the extensive current annealing attempts due to device failure.

S9. Current annealing measurements of CVD graphene device S12D14

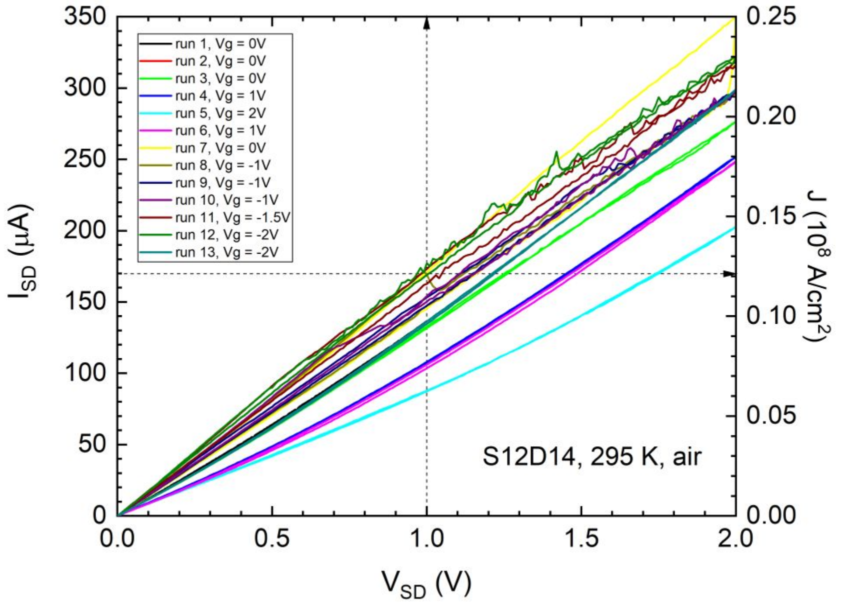

Figure S9. Current annealing characterization of CVDG device S12D14 (L = 2μm, W = 4μm) performed at 295K in air.

S10. Current annealing measurements of CVD graphene device S22D21

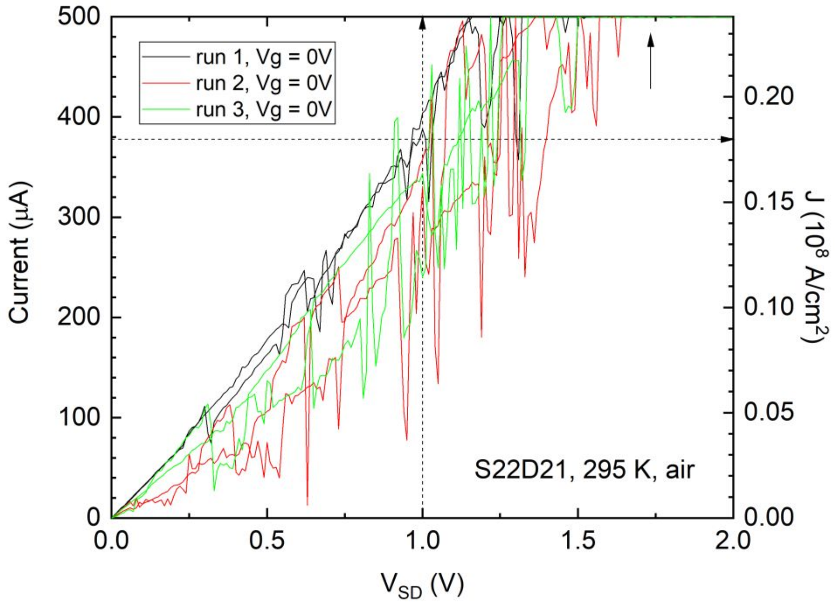

Figure S10. Current annealing characterization of CVDG device S22D21 (L = 2μm, W = 6μm) performed at 295K in air. Note: During the measurement, the current was limited to 500 μA, highlighted using the black arrow to prevent device degradation. However, FET characteristics could not be collected in S22D21 after the current annealing measurement.

S11. Current annealing measurements of CVD graphene device S21D19

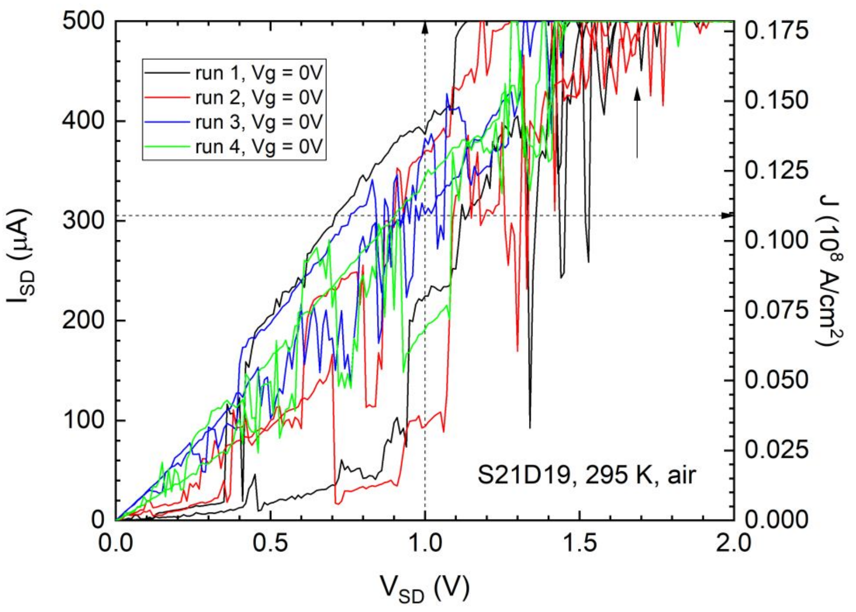

Figure S11. Current annealing characterization of CVDG device S21D19 (L = 2μm, W = 8μm) performed at 295K in air. Note: During the measurement, the current was limited to 500 μA, highlighted using the black arrow to prevent device degradation.
